# News and Coming Events

**Published:** 1908-10-17

**Authors:** 


### News and Coming Events.

Mr. H. Watson Turner, M.R.C.S., L.R.C.P., L.D.S., R.C.S., Eng., has been appointed assistant dental surgeon to the Middlesex Hospital.

Mr. Cyril Maude has lent the Playhouse for November 26 for a matinee in aid of the Infants' Hospital, Vincent Square, Westminster, to be arranged by Mrs. Alfred Mond.

The Royal Hospital for Incurables, Putney Heath, has sustained a serious loss by the death of Mr. J. M. Rucker, Deputy-Chairman of the Institution, which occurred at his residence, Forest Lodge, Putney, on October 13.

Surgeon-Major Thomas Holmested, late Indian Medical Service, died at Weston-super-Mare on September 29. He took part in operations against the Bheels in 1868, having medical charge of the field force employed in Rewa Kanta. He retired in 1878, and then practised at Hyderabad, where he became widely known both as a surgeon and a physician, while in private life he was much esteemed for his charitable deeds.

At the Leeds General Infirmary on October 8, the Lord Mayor, Sir Wilfred Hepton, opened a new ward, which has been provided under a bequest of £124,000 left by Mr. C. S. Weatherill. This, it is stated, forms the most munificent gift of the kind ever received by Leeds. The late Mr. Weatherill, who was a retired provision merchant of the city, left the whole of his fortune, with the exception of £300 a year for his sister, to the Infirmary.

H.R.H. the Princess of Wales opened "The Duchess of Albany Ward" at the Royal Waterloo Hospital for Children and Women, in Waterloo Road, on Saturday, October 10. Upon arriving at the hospital the Princess of Wales was received by the Lord Mayor, who is the president of the institution. Her Royal Highness was presented with an address before declaring the ward open. At the close of the ceremony H.R.H. the Duchess of Albany presented to the Princess of Wales a purse containing contributions. The new ward brings the number of beds in the hospital up to 30, and the number of cots to 60. At the conclusion of the ceremony their Royal Highnesses made a tour of inspection through the various wards of the hospital.

Dr. Eurich, bacteriologist to the Anthrax Investigation Board in Bradford, in his report gives the results of a large number of tests and experiments which he has made with regard to the washing and disinfection of blood-stained material. His conclusions in brief are, first, that blood-stained wool or hair is the carrier of the anthrax bacillus; second, that dust may prove dangerous by virtue of the brittle scales of dried blood-clots derived from such tainted material; third, that a fallen fleece is dangerous in the measure in which it is blood-stained, and that pieces of wool or hair, if blood-stained, may be dangerous. The Lord Mayor of Bradford, Mr. J. E. Fawcett, who is chairman of the Anthrax Investigation Board, at a meeting of that body held this week, said the report was a remarkable one. The investigation had led to a knowledge vastly in excess of what they had anticipated, and pointed in a direction they had not expected. It proved that the principal carrier of anthrax was blood, so that whenever blood was seen it might be regarded as a danger signal. Steps should be taken to secure that sorters and others might readily recognise bloodstained material, and that the material should either be disinfected or destroyed. The board recommended that every effort should be made to remove and destroy blood-stained material.

Dr. F. W. Lamb has resigned his post as Assistant Lecturer in Physiology at University College, Cardiff, on his appointment as Senior Demonstrator in Physiology at Victoria University, Manchester.

Lieut.-Col. C. H. Melville, M.B., has been appointed a professor at the Royal Army Medical College in the place of Lieut.-Col. A. M. Davies, retired. Lieut.-Col. Melville entered the service as surgeon in January 1886, and was with the Hazara expedition in 1888, receiving a medal with clasp.

Dr. Theodore Günther, the oldest medical officer in Middlesex, has just relinquished his public appointments owing to ill-health. For nearly half a century he has been Poor-law medical officer for Hampton Wick, and he has also held the positions of medical officer of health for Hampton Wick and Teddington since those parishes have enjoyed local self-government.

Professor Lannelongue, in a communication to the Académie des Sciences, reports upon the treatment of human phthisical patients with his serum, which was previously subjected to prolonged experiments on guinea-pigs. According to Reuter's Agency excellent results have been obtained, the serum being administered without the slightest danger and having practically in every case produced a substantial improvement in the patient's condition.

With the view of increasing the public utility of the collection of specimens contained in the museum of the Royal College of Surgeons, the council of the college has arranged for a series of demonstrations to be given in the theatre of the college during the present session. The demonstrations will be given by the conservator, Professor Keith, and by the pathological curator, Professor Shattock. Specimens from the museum will be shown, and their bearings on general and surgical pathology discussed. The demonstrations will undoubtedly prove of practical value to medical practitioners and advanced students. The first demonstration of the series was given by Professor Keith on October 16 at 5 p.m., that being the 115th anniversary of the death of John Hunter, the founder of the museum. His subject was the "Specimens Illustrating Malformations of the Rectum and Anus." Professor Shattock will give the second demonstration on Monday, October 19, at 12 noon, on "Hypertrophy."

The annual observance of Hospital Saturday takes place throughout London, for the 35th time in successive years, to-day, Saturday, October 17. In place of the abolished street collection, special contributions will be given in furtherance of the work of the medical charities. The co-operation has again been secured of the employés of the Post Office, Woolwich Arsenal, the several railway companies, and metropolitan borough councils, as well as that of members of the Metropolitan and City Police, and of friendly, trade, and temperance societies. The ordinary weekly collections, which constitute the principal source of the income of the fund, have already amounted during the current year to about £15,000, being more than £1,000 in excess of the sum during the corresponding portion of last year. Of the £27,140 collected in 1907, the sum of £24,437 was divided among 208 institutions, the general and special hospitals receiving £16,998. The management expenses of the fund last year represented 8.70 per cent. of the gross receipts, as compared with 9.65 per cent. in the previous twelve months.

